# A pharmacist-led medication review service with a deprescribing focus guided by implementation science

**DOI:** 10.3389/fphar.2023.1097238

**Published:** 2023-01-30

**Authors:** Nada Alaa Eddine, James Schreiber, Ahmed F. El-Yazbi, Haya Shmaytilli, Mohamed Ezzat Khamis Amin

**Affiliations:** ^1^ Faculty of Pharmacy, Beirut Arab University, Beirut, Lebanon; ^2^ School of Nursing, Duquesne University, Pittsburgh, PA, United States; ^3^ Department of Pharmacology and Toxicology, Faculty of Pharmacy, Alexandria University, Alexandria, Egypt; ^4^ Faculty of Pharmacy, Alamein International University, El Alamein, Egypt

**Keywords:** implementation science (MeSH), pharmacy, deprescribing, polypharmacy (MeSH), patient satisfaction (MeSH), older adult patients, Lebanon, free medication

## Abstract

**Background:** Little research addressed deprescribing-focused medication optimization interventions while utilizing implementation science. This study aimed to develop a pharmacist-led medication review service with a deprescribing focus in a care facility serving patients of low income receiving medications for free in Lebanon followed by an assessment of the recommendations’ acceptance by prescribing physicians. As a secondary aim, the study evaluates the impact of this intervention on satisfaction compared to satisfaction associated with receiving routine care.

**Methods:** The Consolidated Framework for Implementation Research (CFIR) was used to address implementation barriers and facilitators by mapping its constructs to the intervention implementation determinants at the study site. After filling medications and receiving routine pharmacy service at the facility, patients 65 years or older and taking 5 or more medications, were assigned into two groups. Both groups of patients received the intervention. Patient satisfaction was assessed right after receiving the intervention (intervention group) or just before the intervention (control group). The intervention consisted of an assessment of patient medication profiles before addressing recommendations with attending physicians at the facility. Patient satisfaction with the service was assessed using a validated translated version of the Medication Management Patient Satisfaction Survey (MMPSS). Descriptive statistics provided data on drug-related problems, the nature and the number of recommendations as well as physicians’ responses to recommendations. Independent sample t-tests were used to assess the intervention’s impact on patient satisfaction.

**Results:** Of 157 patients meeting the inclusion criteria, 143 patients were enrolled: 72 in the control group and 71 in the experimental group. Of 143 patients, 83% presented drug-related problems (DRPs). Further, 66% of the screened DRPs met the STOPP/START criteria (77%, and 23% respectively). The intervention pharmacist provided 221 recommendations to physicians, of which 52% were to discontinue one or more medications. Patients in the intervention group showed significantly higher satisfaction compared to the ones in the control group (*p* < 0.001, effect size = 1.75). Of those recommendations, 30% were accepted by the physicians.

**Conclusion:** Patients showed significantly higher satisfaction with the intervention they received compared to routine care. Future work should assess how specific CFIR constructs contribute to the outcomes of deprescribing-focused interventions.

## 1 Introduction

Polypharmacy, commonly known as the concomitant daily uptake of five or more medications (Sloane and Zimmerman, 2018; Zechmann et al., 2020), is prevalent among older adults (Khera et al., 2019; Vasilevskis et al., 2019). With some chronic conditions, the use of multiple medications is essential for the improvement of a patient’s health, making polypharmacy appropriate (Duncan et al., 2017; Mair and Fernandez-Llimos, 2017; Halli-Tierny A et al., 2019). In other circumstances, drug-related problems (DRPs) may occur. According to the Pharmaceutical Care Network Europe (PCNE), a DRP is “an event or circumstance involving drug therapy that actually or potentially interferes with desired health outcomes” (Pharmaceutical Care Network Europe, 2003). When DRPs occur while a patient is using multiple medications, the potential detriment of medications may exceed their projected benefit, deeming polypharmacy inappropriate (Chau et al., 2016; Duncan et al., 2017; Martin et al., 2018). Such DRPs are more likely to occur with Potentially Inappropriate Medications (PIMs). The American Geriatrics Society (AGS) Beers Criteria for Potentially Inappropriate Medication (PIM) use in older adults is an explicit list of PIMs that should be avoided among older adults and patients with certain diseases, prescribed at a lower dose or with caution (Samuel, 2015). When a decision is made to prescribe them, they should be carefully monitored. Beers Criteria PIMs have been found to be associated with poor health outcomes, including confusion, falls, and mortality (Fick et al., 2006; Stockl et al., 2010).

Medication review is often recommended to optimize medication use. Medication review, as an overarching term, is used to describe a review of medicines carried out when a health professional meets a patient and there is a decision to prescribe or stop a medicine following a comprehensive and structured process supported by the patient’s records (National Institute for Health and Care Excellence, 2015). Medication optimization builds on the medication review process and occurs when a patient’s medications have been “optimized” by the health professional care team and, consequently, the patient uses the regimen in an ideal manner to improve health outcomes (McFarland et al., 2021). Thus, a medication review is considered part of the plan for inappropriate therapy resolution aiming for medication optimization (de Oliveira Santos Silva et al., 2019).

One medication optimization strategy that has been gaining momentum in the past few years to address medication safety issues is deprescribing (Bleidt, 2019; Clark et al., 2020; Korenvain et al., 2020). Deprescribing is taken to mean more than simply stopping medicines and is considered to be “a planned, stepwise process, specifying the type of medication in question, detailing explicit goals, and including dose reduction and substitution” (Duncan et al., 2017). Deprescribing is the tapering of medications and has to take place to minimize medication-caused harm and improve outcomes (Bleidt, 2019; Tandun et al., 2019; Farrell et al., 2020). To that end, deprescribing has the potential not only to solve DRPs but also to minimize health costs and improve quality of life, while maintaining or even improving clinical outcomes. Studies addressing deprescribing showed a significant potential for reducing the use of potentially inappropriate medications and subsequent adverse outcomes reduction in various settings (Kojima et al., 2012; Mckean et al., 2016; Cossette et al., 2017; Kimura et al., 2017; Wouters et al., 2017; Vasilevskis et al., 2019; Balsom et al., 2020; Dharmarajan et al., 2020; Kua et al., 2020; Schapira et al., 2020; McCarthy et al., 2022). Deprescribing-focused interventions showed promising results in the available literature. Studies addressing deprescribing in the hospital setting showed a significant decrease in PIMs use among older adults (Mckean et al., 2016; Cossette et al., 2017; Kimura et al., 2017; Vasilevskis et al., 2019; Schapira et al., 2020). Similarly, with five studies conducted in nursing homes, physicians’ or pharmacists-directed reviews led to PIMs and subsequent adverse outcomes reduction (Kojima et al., 2012; Wouters et al., 2017; Balsom et al., 2020; Dharmarajan et al., 2020; Kua et al., 2020). A recent intervention focusing on general practitioners in primary care showed a significant decrease in medications they originally prescribed to patients reducing unnecessary medication use by patients (McCarthy et al., 2022). Deprescribing of specific medications has been investigated in earlier interventions. Many of those showed positive outcomes as those targeting benzodiazepines, antidepressants, antidiabetics, anticholinergics, proton pump inhibitors, and non-steroidal anti-inflammatory medications (Reeve et al., 2017; Maund et al., 2019; Tandun et al., 2019; Martinez et al., 2020; Rashid et al., 2020); while deprescribing of other medications continues to be challenging. For example, the withdrawal of urate-lowering drugs was associated with the recurrence of gout episodes (Beslon et al., 2018). The discontinuation of some preventative medicines, such as warfarin, was found to cause harm (Narayan and Nishtala, 2017). Further, little evidence is known about statins’ maximum duration of use, hence a physician’s clinical judgment is needed to decide whether a patient needs to continue taking a statin (van der Ploeg et al., 2020).

To be successfully implemented in practice, and for this success to be sustainable, some issues should be addressed. Medication reviews successful in achieving medication optimization through deprescribing would involve health professionals of multiple backgrounds. Strategies and interventions that would facilitate this collaboration would include factors such as mutual acceptance and readiness of team members towards collaboration, performing as a team rather than an individual; communication strategies among clinicians and shared decision-making, and care coordination (Mustafa Sirimsi et al., 2022). Further, pharmacist-led deprescribing strategies would need to be conceptualized and guided by implementation science (IS) (Pereira et al., 2021; Ailabouni et al., 2022)—the study of the incorporation of evidence-based findings into practice, to improve healthcare—(Bauer et al., 2015; Ronquillo et al., 2018). One framework that has shown promise in implementing medication regimen optimization services in practice is the Consolidated Framework for Implementation Research (CFIR) (Shoemaker et al., 2017; Baumgartner et al., 2020). The prospect of utilizing CFIR in implementing health services in low- and middle-income countries is promising (Ojo et al., 2021). A recent study applied the CFIR in medication reconciliation implementation in a Brazilian hospital indicating that available resources and communication are key constructs of influence in the implementation process (Fernandes et al., 2022). Further, Shoemaker et.al highlighted the importance of using CFIR in implementing professional services at the community pharmacy level (Shoemaker et al., 2017).

Patients’ attitude toward deprescribing was assessed in multiple studies and a positive attitude was observed in most of these studies (Tegegn et al., 2018; Kua et al., 2019; 2020; Shrestha et al., 2021). For instance, in a study performed in a resource-limited setting in Ethiopia, 82% of patients were willing to have one of their medications stopped if the physician suggested it. Assessing patient satisfaction with pharmacist-led services including medication reviews and medication optimization is increasingly investigated in research (Moczygemba et al., 2010; Kim et al., 2016; Cardosi et al., 2018; Nigussie and Edessa, 2018; Basheti et al., 2019; Jordan et al., 2021; Kabba et al., 2021; Kebede et al., 2021). A recent systematic review showed high patient satisfaction toward pharmacist-led medication review interventions (Bou Malham et al., 2021). In addition, patients showed a positive attitude toward pharmacists’ competencies and involvement in deprescribing, especially the elderly (Bužančić et al., 2021). Further, a study reported that patient satisfaction could be enhanced by deprescribing as patients felt uncomfortable with the use of multiple medications (Reeve et al., 2014). For example, review tackling patient satisfaction with the use of proton-pump inhibitors indicated that patients were more satisfied when taking PPI on-demand rather than chronically, meaning that they showed satisfaction with PPI deprescribing (Boghossian et al., 2017). To that end, assessing patient satisfaction with a pharmacy service after being introduced to a medication review service focusing on deprescribing would be helpful when disseminating and implementing such a service.

This study addresses several literature gaps. First, little research has addressed interventions focusing on deprescribing in facilities serving patients of low socioeconomic status, especially in the primary care setting (Milos et al., 2013; Cheong et al., 2018; Hailu et al., 2020). Patients of low income, a class representing a significant proportion of the global patient population, are most likely to benefit from deprescribing in multiple ways. Those include therapeutic as well as economic benefits resulting from the reduced burden of money spent on medications by institutions that subsidize those medications ensuring the sustainability of service provision to those patients. This is particularly relevant in the Lebanese healthcare system where a significant percentage of medication expenses are not covered. Second, few studies have utilized an implementation science conceptual framework to guide the implementation of interventions with a focus on deprescribing. This approach would ensure that different factors influencing implementation are being considered and incorporated as needed. Third, little work has assessed patient satisfaction related to deprescribing, a key desired outcome of deprescribing interventions that would enhance the incorporation of patient’ preferences in the care process. Finally, few studies addressed deprescribing in the Arab region and in developing countries where polypharmacy is suspected to be as high as it is in developed countries (Alsuwaidan et al., 2019; Al-Dahshan et al., 2020; Badawy et al., 2020; Abu Farha et al., 2021).

This study is guided by the Consolidated Framework for Implementation Research (CFIR), a conceptual framework developed to guide the systematic assessment of implementation contexts while addressing factors that might influence intervention implementation and effectiveness (Shoemaker et al., 2017). The CFIR includes five major domains (intervention characteristics, outer setting, inner setting, characteristics of individuals, and process) with underlying constructs that can potentially influence the implementation of interventions. Using tools such as STOPP/START criteria, this study aimed to develop a pharmacist-led medication review service with a deprescribing focus in a healthcare facility serving patients of low-income receiving medications for free in Beirut, Lebanon followed by an assessment of the recommendations’ acceptance by prescribing physicians. As a secondary aim, the study evaluates the impact of this medication review service on the satisfaction of the study participants compared to the routine care they receive.

## 2 Methods

### 2.1 Ethical consideration

This research project followed a prospective experimental study design. Ethical approval for the study was obtained from the Institutional Review Board (IRB) at Beirut Arab University (protocol number 2022-H-0076-P-M-0465). Written informed consent was obtained from all participants assuring that any information provided by the patients is confidential.

### 2.2 Research setting and study population

The research team searched for a site serving patients of low income at subsidized rates or for free in Beirut, Lebanon. This was intended to provide a dual benefit so that in addition to patient benefit from improved health outcomes, which would apply to patients on polypharmacy in different settings, the site might benefit from decreasing the economic burden of unnecessary medication use. Accordingly, a non-governmental charitable association located in Beirut, Lebanon was selected as the intervention site. The healthcare facility provides low-charge consultations and free medications to 1,000 registered patients of low income. The healthcare team consists of 28 physicians, one full-time nurse, and three assistants in addition to a pharmacist, a volunteer nurse, and assistant staffing the pharmacy. In this facility, GPs prescribe medications, but their role is mostly centered around referral to specialty physicians for patients with chronic conditions.

Following two visits by the intervention pharmacist that were intended to introduce the project to the leadership team of the healthcare facility and study the pharmacy setting, a letter was obtained from the facility manager, who is also a general practitioner at the facility, approving the study execution at the facility. The study was then introduced to all specialty physicians and general practitioners who might interact with eligible patients (a total of 17 physicians and general practitioners) to facilitate the endorsement of the project. Those physicians were candidates for following patients in our sample. The rest of the 28 physicians in the facility were of specialties not related to our study focus such as paediatricians for example. The 17 physicians were then provided with a formal letter describing the project plan. Physicians and pharmacy staff were provided with a copy of the STOPP/START criteria that were used as a key tool in the study (Cowan and Riley, 2015; O’mahony et al., 2015).

The study population consisted of patients registered within the facility. Patients were included in the study if they were 65 years or older, met the World Health Organization’s polypharmacy definition of taking five or more medications (World Health Organization, 2019), picked up their medications themselves, and were cognitively capable of participating in the study. A class of patients collects their medications at the facility while being seen by a physician practicing outside the facility. Those patients were not targeted by this work. The intervention focused on patients seen by physicians in the facility so that the clinical pharmacist researcher can follow up on medication review results with physicians practicing at the facility that the clinical pharmacist researcher can access.

### 2.3 Intervention description

The intervention was guided by the adapted CFIR (Shoemaker et al., 2017). The CFIR comprised constructs that were operationalized and adapted into those five domains as follows: 1) *Proposed pharmacy service characteristics-*This domain addresses relative advantage, adaptability, complexity, and cost of the medication review service; 2) *Outer setting*-This domain addresses external influences on the intervention’s implementation including patient needs and resources, cosmopolitanism or the level at which the pharmacy is networked with other pharmacies, peer pressure, and external policies and incentives; 3) *Inner setting-* This domain addresses different characteristics of the implementing facility such as structural characteristics, pharmacy culture, readiness for implementation including leadership engagement, available resources and access to knowledge and information, and the implementation climate including tension for change, compatibility, organization incentives and rewards; 4) *Pharmacy staff characteristics*- This domain addresses pharmacist’s and pharmacy staff’s beliefs about the intervention, self-efficacy, and other personal attributes that may affect implementation; 5) *Process of implementation*- This domain addresses stages of implementation for the proposed pharmacy service such as planning, engaging formally appointed internal leaders, champions and external change agents executing, reflecting and evaluating the intervention. A detailed mapping of the intervention setup to the CFIR is described in Table 1. The intervention was first tested on a convenience sample of eight patients at the facility using less than five medications. This was followed by a pilot study of 46 participants meeting inclusion criteria including the use of five or more medications. Findings from pilot study participants meeting the inclusion criteria were included in the final calculations since no changes were made to the employed methods following this step. Data collection for the pilot and the full study took place between June and September 2021.

**TABLE 1:** The deprescribing intervention setup mapped to the adapted Consolidated Framework for Implementation Research.

Construct	Subconstruct	Description	
Pharmacy Service Characteristics	Relative advantage	**Perception of staff, providers, and patients of the benefits of deprescribing services.**	
		The intervention pharmacist met with the facility manager and explained the benefits of providing deprescribing services for patients at the facility. The perception of the facility staff towards the intervention was favorable. To address staff perceptions of the project, the intervention pharmacist drafted a letter to the facility physicians explaining the rationale for the project and provided them with a description of the materials to be used including a copy of the STOPP/START criteria, after getting the acceptance of those physicians to be involved in the study.	
	Adaptability	**The degree to which deprescribing service can be tailored to meet local needs at the organization.**	
		The deprescribing service meets the local needs of the organization by 1) improving patient outcomes, and 2) reducing the cost of inappropriate medications that burden the budget of the facility at a time of an economic crisis**.**	
	Complexity	**The difficulty of implementing deprescribing service (scope, incorporation into workflow, staff needed, number of steps required).**	
		The intervention pharmacist shadowed the pharmacy staff before proposing the intervention, which was piloted to ensure smooth integration into the workflow, without being staff intensive and to improve the feasibility and patient safety.	
	Cost	**Cost of providing deprescribing service.**	
		The deprescribing intervention was provided by the intervention pharmacist for no charge and was not sponsored by extramural funds.	
Outer Setting	Patient needs and resources	**A patient’s needs for deprescribing; barriers and facilitators to meet these needs.**	
		A significant proportion of patients getting their services from the facility are on polypharmacy. This puts the patients in need of intervention.	
		The pharmacy staff members do not have the time to perform additional cognitive services. Therefore, the deprescribing service provided by a pharmacist from the outside would complement the routine counseling that is currently provided to patients. The intervention pharmacist was supported by two data collectors who were members of the research team.	
	Cosmopolitanism	**The degree to which a pharmacy is networked with other pharmacies and providers.**	
		There is only one pharmacy at the facility and it was not networked with external pharmacies. Still, a connection was made between the intervention pharmacist and the providers at the facility as described in the intervention flow and description part.	
	Peer Pressure	**Competitive pressure to provide a deprescribing service.**	
		Since this is a charity organization, there is little competitive pressure to introduce a new service.	
	External policy and incentives	**Strategies to spread deprescribing services, including policy and regulations, external mandates, recommendations and guidelines, pay-for-performance, collaboratives, and public and benchmark reporting.**	
		The facility management had a dual incentive in the deprescribing intervention: 1) improved patient outcomes, 2) reduced cost of unhelpful medications that burden the budget of the facility at a time of economic crisis.	
Inner Setting	Structural characteristics	**Type of the pharmacy, size, physical space, staffing**.	
		A small-sized (420 cm*600 cm) pharmacy, located inside the organization, is managed by a pharmacist, a nurse, and an assistant. The pharmacy does not have a designated counseling area due to its relatively small size. However, a space at the facility next to the pharmacy was offered for the intervention pharmacist to interview patients.	
	Culture	**Norms, values, and basic assumptions of the pharmacy.**	
		The intervention fits within the values assessed in the facility, which emphasized providing high-quality services for vulnerable patients at little to no cost.	
	Implementation climate	Tension for change	**The degree to which the facility manager and staff perceived the situation as needing change and that a deprescribing service should be provided.**
			The manager of the facility and the site pharmacist agreed that their patients need the proposed intervention.
		Compatibility	**Degree of tangible fit between meaning and values attached to deprescribing service by pharmacy staff.**
			The mission of the institution as a charitable organization increased the enthusiasm of the facility manager and staff members to adopt the intervention.
		Organizational incentives and rewards	**Awards, performance reviews, promotions, raise in salary, increased stature, or respect.**
			The facility has not adopted such policies at the time of the intervention.
	Readiness for implementation	Leadership engagement	**Commitment, involvement, and accountability of leaders and pharmacy managers in implementing and providing a deprescribing service.**
			The facility manager, the executive health officer, and the pharmacy staff manager were committed to the intervention's success. The manager of the facility fully approved the study plan and participated in the study as one of the study physicians. A support letter was provided to the intervention pharmacist.
		Available resources	**Money, training, education, physical space, and time dedicated to providing deprescribing.**
			A private place adjacent to the pharmacy was provided to the intervention pharmacist for patient interviewing. The intervention pharmacist has provided enough time for completing the deprescribing service.
		Access to knowledge and information	**Ease of access to digestible information and knowledge about deprescribing services.**
			The updated guidelines for each medical condition, the last version of the STOPP/START criteria, and the Medscape drug interaction checker were available and utilized by the intervention pharmacists when needed. Recommendations from the intervention pharmacist were double-checked by a licensed pharmacist who is also a professor of pharmacotherapeutics before presenting these recommendations to physicians.
Pharmacy staff Characteristics	Knowledge and beliefs about the intervention	**Pharmacy staffs’ attitudes, values, and familiarity with deprescribing components, steps, documentation, and care process.**	
		The pharmacy staff was introduced to the deprescribing service plan before the intervention commencement by the intervention pharmacist. A documentation form for each patient was provided to the facility manager at the end of the project.	
	Self-efficacy	**Pharmacy staff’s belief in their capabilities to provide deprescribing.**	
		The intervention pharmacist was the one providing the intervention, and the pharmacy staff was not a part of the study.	
	Other personal attributes	**Tolerance of ambiguity, intellectual ability, motivation, values, competence, capacity, and learning style.**	
		Those personal attributes were not examined.	
Process	Planning	**The degree to which a scheme or a method for implementing a deprescribing service is developed and the quality of this scheme**.	
		The intervention was tested and piloted in an extensive process.	
	Engaging	Formally appointed internal leaders	**Individuals from within the pharmacy who have been formally appointed with responsibility for implementing and overseeing deprescribing.**
			The intervention pharmacist was responsible for implementing the intervention at the highest possible standard as part of an agreement with the facility director.
		Champions	**Individuals who dedicated themselves to supporting and driving through the provision of deprescribing.**
			The manager of the facility was the champion of the intervention as he discussed with the prescribing physicians the importance of implementing deprescribing at the facility.
		External change agents	**Individuals who are affiliated with an outside entity formally influence decisions in a desirable direction to provide deprescribing.**
			No external change agents contributed to the intervention implementation.
	Executing	**Carrying out implementation according to plan.**	
		The intervention pharmacist strictly followed the research plan as part of her Master’s research.	
	Reflecting and evaluating	**Feedback about the progress and quality of deprescribing service, with a regular reflection on progress and experience.**	
		The intervention pharmacist regularly debriefed the thesis advisor and the manager of the facility about the progress of the study. A licensed pharmacist was debriefed about each recommendation before being presented to physicians.	

Participants were approached by a trained data collector with a clinical pharmacy background after picking up their medications from the pharmacy at the facility, which included their routine interaction with the facility site pharmacist, who provides routine medication dispensing at the pharmacy. Informed patient consent was made by the intervention pharmacist or a trained data collector with a clinical pharmacy background at this point while explaining to patients that the care they receive at the facility would not be impacted in any way if they choose not to participate. Participants were divided into two groups in order of their exit from the pharmacy. The intervention group was first introduced to the study purpose of deprescribing PIMs, received disease and medication counseling, and was then administered the translated Medication Management Patient Satisfaction Survey (MMPSS) (Moon et al., 2016). The MMPSS was developed with the aim of providing a reliable and brief patient satisfaction survey specific to pharmacists providing comprehensive medication management services. It consists of ten questions, nine of which used a scale from 1 to 4 (strongly agree, agree, disagree, strongly disagree) and asks patients to evaluate their experiences with the clinical pharmacist. The final question asks patients to rate their overall quality of care and services on a Likert-scale from 1 to 5 (excellent to poor). An aggregate scale score for MMPSS is calculated by summing the score for each item in the scale. See Supplementary Appendix SA1.

On the other hand, the control group was administered the MMPSS satisfaction survey before taking the intervention. For ethical reasons, control group patients were provided with the same intervention by the same intervention pharmacist following filling out the MMPSS survey. The satisfaction survey was administered verbally by two trained data collectors.

The patient interview lasted for 15–20 min and consisted of gathering data by the intervention pharmacist addressing demographic and full medical profile information with patients. These data included underlying medical conditions, chronic and acute medication lists including non-prescription and herbal products, any problem with medication, previous medical and surgical history, and family history. Those data were used along with data from the patient’s health file at the facility to determine the current medications the patient is taking without reconciliation. Afterward, each patient’s condition, from both groups, was assessed to screen for DRPs including PIMs and potential prescribing omissions (PPOs) using the most updated version of the STOPP/START criteria as well as the newly updated clinical guidelines of relevance to each case. The STOPP/START criteria are organized according to specific physiological body systems, thereby enhancing their useability. STOPP criteria, in particular, were selected for their comprehensiveness and sensitivity in assessing PIMs compared to alternative explicit criteria (e.g., Beers criteria) (Aguiar et al., 2021). The Medscape interaction checker was utilized in the initial assessment for the compatibility of drugs while checking related guidelines as needed including those by the American College of Cardiology/American Heart Association, the European Society of Cardiology, the Global Initiative for Chronic Obstructive Lung Disease, and the American Diabetes Association (O’Gara et al., 2013; Amsterdam et al., 2014; Yancy et al., 2017; Valgimigli et al., 2018; Whelton et al., 2018; Arnett et al., 2019; Grundy et al., 2019; Vogelmeir et al., 2020; American Diabetes Association, 2021; Medscape, 2021). The clinical pharmacist researcher assigned DRPs to one of eleven categories: no or unclear indication, better alternative available, regimen needs simplification, overdosage, overuse of therapy, drug-drug interaction, presence of an adverse drug reaction or drug allergy, unsafe medication, duplication therapy, omission, regimen needs intensification (Pharmaceutical Care Network Europe, 2003; Lim et al., 2018). Later, therapeutic recommendations which include drug discontinuation, dosage or regimen adjustment, drug substitution, or new drug prescription, were double-checked with a licensed pharmacist who is also a professor of pharmacotherapeutics. Final recommendations were then presented and discussed with prescribing physicians at the intervention site. In some instances, multiple DRPs for a patient would have been solved by one proposed recommendation, so the number of DRPs and the number of recommendations were not expected to match. These recommendations were either accepted, deferred (postponed to monitor the current patient’s condition or to order laboratory testing), or rejected. The acceptance rate of DRPs was analyzed and presented according to physician specialty.

At the end of the project, a discussion was conducted with the manager of the facility to highlight all the recommendations and subsequent physicians’ responses, together with the researcher’s suggestions aimed at optimizing patient care. A copy of each patient documentation form was provided to the facility manager for the sustainability of patient care at the facility.

### 2.4 Study outcomes

The primary outcome of this study was the impact of the medication optimization deprescribing focused intervention on patient satisfaction. The process of translating, adapting, and validating the Medication Management Patient Satisfaction Survey (MMPSS) into Lebanese Arabic is described in the thesis of the first author (Alaa Eddine N, 2022). A manuscript describing the process is currently under review in this journal. Secondary outcome measures were the number of changes and subcategories of changes proposed by the pharmacist including drug discontinuation, substitution, initiation, or dosage adjustment, and the proportion of changes accepted by the prescribing physicians measured as a percentage of accepted, deferred, or rejected recommendations for each physician.

### 2.5 Statistical analyses

Descriptive statistics were used to describe the characteristics of the sample. They also provided data on drug-related problems, the nature and the number of recommendations as well as physicians’ responses to recommendations. Descriptive analyses were done using IBM SPSS 24^®^ generating frequencies, as well as means and ranges as relevant.

Descriptive statistics of the summary score (items 1–9) of MMPSS were computed for each experimental group to assess patient satisfaction with the service. Independent sample t-tests were used to check for the difference between control and intervention groups for the summary score of items one to nine and for item ten alone. Responses for items one to nine were coded from 0 to 3 with “strongly disagree” given a code of 0 and “strongly agree” given a code of 3. For item 10, a similar coding was followed with “poor” given a code of 0 and “excellent” given a code of 4.

A pilot study was carried out on 46 patients (23 per group) to calculate the required sample size needed that would ensure that the study is adequately powered. *A priori* power analysis was done and accordingly, a sample size of 57 patients per group was needed to detect an intervention effect size on patient satisfaction of 0.5 as measured by MMPSS, with a power of 80%. Power analysis and the analysis of the intervention’s impact on patient satisfaction and on were done using the online software Jamovi^®^.

## 3 Results

### 3.1 Participants’ characteristics

**FIGURE 1:**
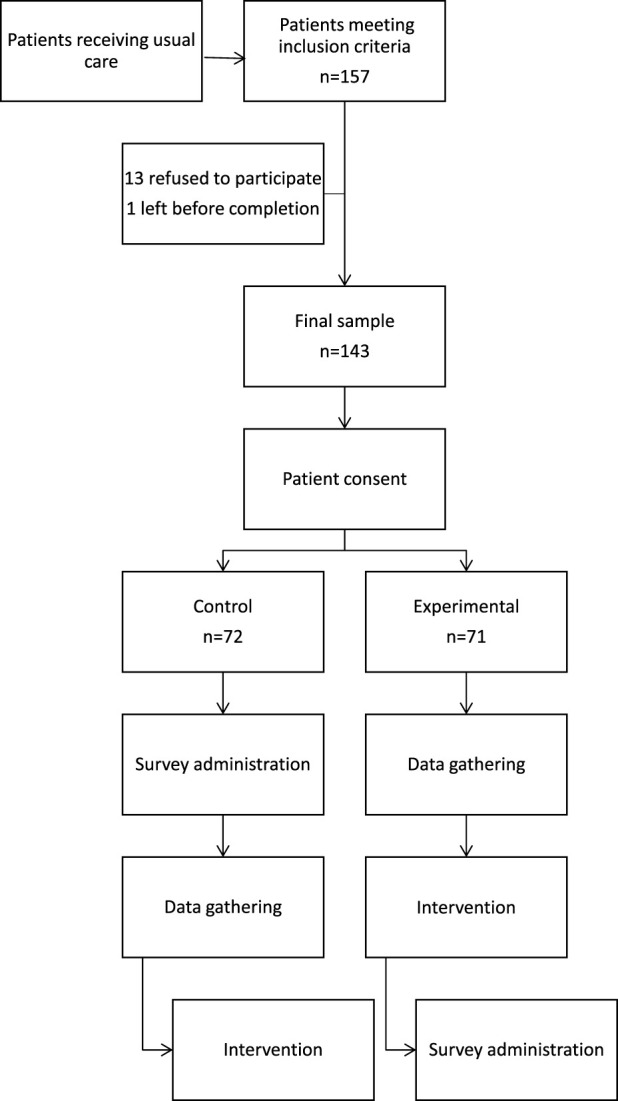
Medication optimization intervention flow diagram.

In total, 157 patients met the inclusion criteria for the study. Of those, 143 patients were enrolled in the study: 72 patients in the control group and 71 patients in the experimental group. See Figure 1. The mean age of the patients was 72 years in both groups. The majority of the patients were female (67% in the control group and 70% in the experimental group). Patients had an average of four comorbidities and were using an average of eight medications daily in both groups. See Table 2.

**TABLE 2:** Participants’ characteristics and descriptive statistics.

		Control	Intervention
		*n* = 72 (50.3%)	*n* = 71 (49.7%)
Age	Mean	72	72
	Range	65–86	65–86
Gender	Female: frequency (%)	48 (67)	50 (70)
Number of comorbidities	Mean	4	4
	Range	1–9	1–8
Number of medications	Mean	8	8
	Range	5–14	5–17

### 3.2 Drug-related problems among the study population

After assessing all patient profiles (72 control and 71 experimental), DRPs were analyzed for both groups and presented for intervention and control groups according to the Anatomical Therapeutic Classification (ATC) classification of medications indicating comparable rates of medication use in different categories. See Table 3. Overall, 25 patients (18%) had no DRPs and 83% had one or more DRPs. Those DRPs ranged from one DRP (44%) to six DRPs per patient (1%), with a total of 231 DRPs. A drug regimen that needs to be intensified counted for the highest percentage (22%), followed by a drug with no or unclear indication (16%), an unsafe medication (16%), and a drug omission (14%). Further, 66% of the screened DRPs met the STOPP/START criteria (77%, and 23% respectively). The most common DRP in the STOPP category was that Aspirin was not indicated; while in the START category, the need for an Angiotensin-Converting Enzyme Inhibitor (ACEI) was the most common DRP encountered. The remaining DRPs were revealed by matching their use to guideline-based recommendations or by running medications used through a drug-interaction checker. See Table 4.

**TABLE 3:** Anatomical Therapeutic Classification (ATC) classification of medications among intervention and control two groups (World Health Organization, 2022).

Medication ATC class	Medications taken by the control group n (%)	Medications taken by the intervention group n (%)	Total n (%)
A: Alimentary tract and metabolism	127 (46)	152 (54)	279 (24)
B: Blood and blood-forming organs	73 (49)	75 (51)	148 (13)
C: Cardiovascular system	271 (51)	259 (49)	530 (46)
G: Genito urinary system and sex hormones	5 (45)	6 (55)	11 (1)
H: Systemic hormonal preparations excluding sex hormones and insulins	21 (43)	28 (57)	49 (4)
M: Musculoskeletal system	16 (52)	15 (48)	31 (3)
N: Nervous system	13 (34)	25 (66)	38 (3)
R: Respiratory system	34 (52)	31 (48)	65 (6)

**TABLE 4:** List and number of drug-related problems (*N* = 231).

Drug-related problem type	Total n (%)	Intervention n (%)	Control n (%)
Regimen needs intensification	50 (21.7)	25 (50)	25 (50)
No or unclear indication	38 (16.4)	15 (39)	23 (61)
Unsafe medication	36 (15.7)	19 (53)	17 (47)
Omissions	33 (14.3)	18 (55)	15 (45)
Overduration of therapy	25 (10.9)	15 (60)	10 (40)
Drug-drug interaction †	23 (10)	15 (65)	8 (35)
Presence of an adverse drug reaction or drug allergy	13 (5.6)	9 (69)	4 (31)
Other DRPs^a^	13 (5.6)	6 (46)	7 (54)
Total	231 (100)	122 (53)	109 (47)

^a^ Those included having a better alternative available, regimen needs simplification, medication overdosage, duplication of therapy, † All drug interactions were assessed using the Medscape interaction checker as serious, except for Amiodarone with Acenocoumarol (not found in Medscape).

### 3.3 Recommendations made to physicians and response to those recommendations

In total, 221 recommendations, divided into six categories, were provided to physicians. Of those recommendations, more than half, 52%, were to discontinue one medication, 23% were to intensify a therapeutic regimen, and 13% were to initiate one medication. Added together, deprescribing recommendations; i.e., discontinue a medication, decrease a dose, switch to an alternative, and simplify a regimen; comprised 64% of recommendations, which was almost double the sum of drug initiation and regimen intensification recommendations (36%). See Table 5. The intervention pharmacist discussed the recommendations with three cardiologists, two endocrinologists, two gastroenterologists, one pulmonologist, and one orthopaedist. The majority of the recommendations were provided to cardiologists (72%). More than half of the total recommendations were rejected (56%), 30% were accepted, and the remaining (14%) were deferred. The rate of accepting recommendations was not uniform across physicians. The pulmonologist and the orthopaedist accepted all the recommendations received, while cardiologist-1 and gastroenterologist-1 showed an acceptance rate of 42% followed by endocrinologist-1 (35%). Cardiologist-3, on the other hand, accepted only 5% of the recommendations provided (Table 6).

**TABLE 5:** List, number and proportion of recommendations made to physicians (*N* = 221).

Recommendation type	n (%)
Drug discontinuation	114 (51.5)
Regimen intensification	50 (22.6)
Drug initiation	29 (12.9)
Drug substitution	18 (8.3)
Regimen simplification	7 (3.3)
Dosage adjustment	3 (1.4)

**TABLE 6:** Number of recommendations and associated responses per physician specialty (*N* = 221).

Physician speciality	Number of recommendations	Accepted Number(%)	Rejected Number(%)	Deferred^a^ Number(%)
Cardiologists	158	44 (28)	94 (59)	20 (13)
Endocrinologists	31	9 (29)	15 (48)	7 (23)
Gastroenterologists	25	7 (28)	15 (60)	3 (12)
Pulmonologist	3	3 (100)	0 (0)	0 (0)
Orthopaedist	4	4 (100)	0 (0)	0 (0)
Total	221	67 (30)	124 (56)	30 (14)

^a^ Deferred means the response of the physician was postponed to a later time to monitor the patient or to order laboratory tests.

### 3.4 Satisfaction with the intervention

Descriptive statistics of the summary medication management patient satisfaction score (MMPSS items one to nine) per group are presented in Table 7. Patients in the control and experimental groups had a meaningful difference in satisfaction in favor of the provided intervention. Cohen’s d effect size of 1.75 (*t* (141) = −10.48, *p* < 0.001). Data in both groups were not normally distributed. Due to the non-normality of the outcome by groups, a Mann-Whitney non-parametric analysis was also completed, providing the same conclusion (Table 8). The control group had a mean score of 15.2 (SD: 3.74), which is statistically significantly lower than that of the experimental group by six points (21.1; SD: 2.9).

**TABLE 7:** Descriptive statistics of the summary medication management patient satisfaction score per group.

	Group^a^	N	Mean	Median	SD	SE	Minimum	Maximum
Summary score	C	72	15.24	15	3.74	0.44	8	27
	I	71	21.11	21	2.91	0.35	14	27

^a^ C:Control Group; I = Intervention Goup.

**TABLE 8:** Difference in patient satisfaction between groups.

		Statistic	Df	P	Mean difference	SE difference		Effect size
Summary score	Student’s *t*	−10.48	141.00	<.001	−5.88	0.56	Cohen’s d	−1.75
	Welch’s *t*	−10.49	133.81	<.001	−5.88	0.56	Cohen’s d	−1.75
	Mann-Whitney U	508.50		<.001	−6.00		Rank biserial correlation	0.80

## 4 Discussion

### 4.1 Key findings

This study assessed the impact of a pharmacist-led medication optimization service with a deprescribing focus targeting older adults of low income on polypharmacy while utilizing the adapted consolidated framework for implementation research in planning the service (Shoemaker et al., 2017). Results showed that problems related to medications and medication inappropriateness are widespread among the studied population providing an opportunity for a pharmacist-driven intervention. While physician acceptance of provided pharmacist recommendations was not optimal, patients provided with this service showed much greater satisfaction with the provided service compared to the regular medication dispensing service routinely provided by the facility site pharmacist.

### 4.2 Drug-related problems and potentially inappropriate medications

Regarding drug-related problems, the findings reported here are in line with other studies that show a high prevalence of DRPs in older adults (68%–93%) (Allard et al., 2001; Sellors et al., 2003; Milos et al., 2013; Chan et al., 2014). Of the reasons that typically cause this high DRP prevalence among the studied population in different settings, the fact that recruited patients were suffering from multiple comorbidities and hence, were followed by multiple prescribers, with insufficient coordination between them might have been a key issue driving DRPs in this patient population (Vinks et al., 2009; Tan et al., 2014; Campbell A et al., 2018; Cheong et al., 2018; Khera et al., 2019). Still, it is important to note that DRPs found in this study at this facility were comparable to the literature as indicated above. This indicates that, despite the limited resources, the quality of patient care in this setting is comparable to others.

Being the main target, PIMs, as defined by the STOPP criteria, counted for half of the DRPs in this study, with the majority of PIMs falling in the cardiovascular drug class (no indication for Aspirin and long-term use of DAPT), followed by the unsafe use of sulfonylurea in elderly. Consistent with our results, studies addressing PIM prevalence among elderly patients indicated a prevalence of PIMs of 45%–60% (Saab et al., 2006; Eze and Olowu, 2011; Zeenny et al., 2017). A study conducted in Ethiopia revealed that the inappropriate use or omission of antithrombotic medications is prevalent in Ethiopian older adults (Getachew et al., 2016). Other studies determined PPIs, antithrombotic, sulfonylurea, and benzodiazepines as the most frequent PIMs screened by STOPP criteria (Dalleur et al., 2014; Chau et al., 2016; Kimura et al., 2017), in addition to NSAIDs, skeletal muscle relaxants, antihistamines, estrogen, and drugs for the central nervous system, as described by Beer’s criteria (Fadare et al., 2013; van Heerden et al., 2016; Ammerman et al., 2019; Cardwell et al., 2020; Deyo et al., 2020) This diversity in reported PIMs is common among studies. In this study, patients were of low income, hence, OTC medications (NSAIDs, antihistamines), and other medications not provided by the facility (CNS drugs) were not likely to be used, to begin with, as they were not typically affordable to patients. Second, patient records that were available at the facility for the intervention pharmacist might have been missing some information on the use of those products.

### 4.3 Clinical pharmacist’s recommendations and physician acceptance

The recommendations provided by the intervention pharmacist to the specialty physicians at the facility mainly focused on deprescribing rather than initiating new medications, with the most common being discontinuing a medication. Intensification of a regimen, on the other hand, was the second common recommendation. This comes in agreement with literature where drug discontinuation was a frequent recommendation in many pharmacist-led interventions targeting elderly patients on polypharmacy (Vinks et al., 2009; Milos et al., 2013; Chau et al., 2016; Campins et al., 2017; Hurmuz et al., 2018; Cardwell et al., 2020). Some studies described pharmacist recommendations that only focused on providing deprescribing recommendations to physicians without addressing therapy intensification. (Kurt Kroenke and Pinholt, 1990; Dalleur et al., 2014; Morrison and MacRae, 2015; Pruskowski and Handler, 2017; Wouters et al., 2017; Cheong et al., 2018; Clark et al., 2020; Deyo et al., 2020; Kua et al., 2021). On the contrary, drug initiation, patient education, laboratory monitoring, and dose adjustment were more common recommendations in other studies (Tan et al., 2014; Campbell A et al., 2018; Khera et al., 2019). One of the latter studies targeted inappropriate medication use among elderly patients without necessarily being on polypharmacy (Campbell A et al., 2018). In addition, the explicit criteria for screening for PIMs such as the STOPP/START criteria were not used in some of these studies (Tan et al., 2014; Campbell A et al., 2018). These factors could all result in differences in pharmacists’ recommendations between studies.

Thirty percent of the recommendations provided to different specialty physicians were accepted in this study. This acceptance rate is in line with several studies performed in multiple settings (20%–42%) (Vinks et al., 2009; Touchette et al., 2012; Pruskowski and Handler, 2017; Clark et al., 2020). Higher acceptance rates (75%–99%), however, were observed in other studies (Blakey and Hixson-Wallace, 2000; Roth et al., 2013; Campins et al., 2017; Kimura et al., 2017; Cheong et al., 2018; Balsom et al., 2020; Kua et al., 2021). Hailu et.al, a study conducted in a hospital in Ethiopia, showed a similar rate of DRPs (82%), but a high acceptance rate (92%) (Hailu et al., 2020). The fact that many of the drugs that constituted better alternatives for patients were in shortage at the time of the study has likely contributed to a significant proportion of those physician rejections for the intervention pharmacist recommendations. Under conditions of resource scarcity, physicians tend to give patients the available medication, even if it does not provide the most optimal therapeutic effect, rather than keeping the patient without therapy. This issue is often overlooked in research that is carried out in settings where resource scarcity does not represent a significant barrier and would warrant exploration in future work. Even with this factor taken into consideration, it was interesting to note that adopting an implementation science approach in planning this study did not seem to increase the acceptance rate of physicians in this setting above average rates reported in the literature. This suboptimal acceptance rate for recommendations provided by pharmacists should be explored in the future implementation of science-driven interventions building on and complementing this work.

### 4.4 Patient satisfaction with the intervention

The fact that patients receiving services in the facility welcomed the intervention provided by the intervention pharmacist is a key finding in this study. One would have suspected that patients, in this kind of setting, where medications are provided free of charge may have received an intervention focusing on a reduction in the number of offered medications with suspicion. This did not seem to be the case with the intervention producing a wide margin of increase in patient satisfaction. This finding could be promising with regards to patient satisfaction towards deprescribing in settings providing medications for little to no charge and requires further investigation. It is possible that because START/STOPP criteria were used, patients felt they would get additional medications prescribed as a result of their medication review, not just have medications deprescribed, in case their clinical condition required so. This could have further boosted their satisfaction with the provided service and reduced the likelihood of the misconception that the economic saving from deprescribing medications was the key driver for the work.

Earlier research assessed patient satisfaction with pharmacist-provided care in the community, primary care, and hospital settings (Alhomoud et al., 2016; Soeiro et al., 2017; Nigussie and Edessa, 2018; Jordan et al., 2021; Kabba et al., 2021; Kebede et al., 2021). In most of these studies, research indicated that patient satisfaction with pharmacist services was linked to interpersonal aspects such as communication with and being respectful to patients, as well as disease and therapy management offered by pharmacists. In Sierra Leone and Ethiopia, where health resources are relatively limited, an important factor leading to patient satisfaction was having those services provided by the pharmacist for free (Kabba et al., 2021; Kebede et al., 2021). This is in line with our study, where the intervention pharmacist reviewed therapeutic regimens for patients of low income and completed the service for no charge.

An implementation science approach was used to guide the provision of the pharmacist intervention in this study. The CFIR, which was applied here, was previously used in implementing various health interventions such as the implementation of fall prevention projects, clinical practice guidelines in nursing practice, and the HPV vaccine schedule among adolescents (Shaw et al., 2013; Breimaier et al., 2015; Garbutt et al., 2018). Taken together and in agreement with this study, these findings indicate that the application of CFIR proved to be a helpful framework in organizing the implementation process of those projects being particularly valuable in managing barriers and facilitators behind the success of the implementation.

Further, the literature indicates that patient satisfaction with health interventions guided by implementation science is promising. The application of implementation science in family planning and in providing life narrative interviews for medical inpatients led to high satisfaction and acceptance among participants (Rybarczyk et al., 2019; Weis and Festin, 2020). Moreover, an initiative to implement a measurement program of office and home blood pressure in primary care demonstrated favorable satisfaction among patients and providers towards the service that might decrease the use of unnecessary antihypertensive medications and enhance hypertension control (Doane et al., 2018). These findings are consistent with our study, where patient satisfaction with the provided intervention that was guided by implementation science was high.

### 4.5 Implications for practice

This study calls for increased attention to this population in future research and targeted medication optimization interventions. Pharmacists and other health professionals should be proactive in pursuing deprescribing-focused interventions in settings with limited resources noting the benefits of such interventions in these settings including increased patient satisfaction. In countries where deprescribing has not been integrated into practice, as in Lebanon, measures could be taken to facilitate the transition using an implementation science approach that considers as many implementation science considerations as possible. This includes providing facilities with different resources for intervention success; education and training of physicians, pharmacists, and medical staff; together with the incorporation of deprescribing guidelines and tools such as the STOPP/START criteria into routine practice. It is also recommended that mechanisms of financing deprescribing related activities would be established to promote its sustainability. These mechanisms should be informed by cost effectiveness analyses of interventions such as this, which are typically done and presented separately. This would be interesting to pursue and could be investigated in future research.

Another area for future research would consider the benefits vs. risks of deprescribing medication using tools such as STOPP/START criteria accounting for risks such as rebound of conditions as a result of the withdrawal of specific medications. Clinicians would benefit from extra training on managing those risks as part of different aspects to effectively and safely implement deprescribing. This along with the facilitation of interprofessional therapeutic management of medication regimens would lead to better patient satisfaction and outcomes.

### 4.6 Strengths and limitations

This prospective experimental study has specific strengths. It is unique in coupling the deprescribing approach with the use of an implementation science framework in planning the intervention while assessing patients’ satisfaction with the medication management pharmacist’s service. Still, this study had its limitations. It was conducted in a single center in Lebanon, limiting its generalizability. In addition, for logistical reasons, the implementation and outcomes of recommendations were not monitored. Studies spanning those areas could be targeted by future research.

## 5 Conclusion

This intervention conducted in a facility serving patients of low income found a high prevalence of inappropriate medications taken by these patients comparable to the literature indicating that, despite the limited resources, the quality of patient care in this facility is comparable to other settings. Further, in a facility where medications are provided free of charge, patients enrolled in the study were highly satisfied with the new service they received from the pharmacist showing promise for future interventions addressing deprescribing in similar settings. The pharmacist performing the intervention provided suggestions to physicians yielding an average acceptance rate, which calls for further research into the best ways of integrating implementation science principles in guiding pharmacist interventions. Future work should assess how specific CFIR constructs contribute to the outcomes of deprescribing interventions.

## Data Availability

The raw data supporting the conclusion of this article will be made available by the authors, without undue reservation.
